# MEMS Resonant Beam with Outstanding Uniformity of Sensitivity and Temperature Distribution for Accurate Gas Sensing and On-Chip TGA

**DOI:** 10.3390/s24082495

**Published:** 2024-04-13

**Authors:** Zheng Lu, Hao Jia, Ding Wang, Haitao Yu

**Affiliations:** 1 School of Materials and Chemistry, University of Shanghai for Science and Technology, Shanghai 200093, China; 212203137@st.usst.edu.cn (Z.L.); wangding@usst.edu.cn (D.W.); 2 State Key Laboratory of Transducer Technology, Shanghai Institute of Microsystem and Information Technology, Chinese Academy of Sciences, Shanghai 200050, China; hao.jia@mail.sim.ac.cn

**Keywords:** resonator, microcantilever, resonant beam, uniformity, gas sensing, TGA

## Abstract

Micromechanical resonators have aroused growing interest as biological and chemical sensors, and microcantilever beams are the main research focus. Recently, a resonant microcantilever with an integrated heater has been applied in on-chip thermogravimetric analysis (TGA). However, there is a strong relationship between the mass sensitivity of a resonant microcantilever and the location of adsorbed masses. Different sampling positions will cause sensitivity differences, which will result in an inaccurate calculation of mass change. Herein, an integrated H-shaped resonant beam with uniform mass sensitivity and temperature distribution is proposed and developed to improve the accuracy of bio/chemical sensing and TGA applications. Experiments verified that the presented resonant beam possesses much better uniformity of sensitivity and temperature distribution compared with resonant microcantilevers. Gas-sensing and TGA experiments utilizing the integrated resonant beam were also carried out and exhibited good measurement accuracy.

## 1. Introduction

There is increasing interest in micromechanical resonators as sensors for biological and chemical applications, which correlate the mass of adsorbed materials to shifts in the sensor resonance frequency [1,2,3,4,5]. Among these resonators, resonant microcantilever beams, which originated from scanning probes in atomic force microscopy (AFM) and were pioneeringly used for gas detection by Thomas Thundat et al. at the Oak Ridge National Laboratory in 1995 [6], are the main research focus. Recently, a resonant microcantilever with an integrated heater has been presented and utilized in on-chip thermogravimetric analysis (referred to as MEMS TGA) [7]. TGA is a classic characterization method in many application fields, such as new material development, chemistry, physics, and energy [8,9,10,11,12]. The core measuring component of traditional TGA instruments is a bulky thermobalance. Replacing the thermobalance with the resonant microcantilever, MEMS TGA exhibits significant advantages over traditional TGA, including minimal sample amount requirement, ultra-high-resolution mass measurement, and extremely high ramp rate and analysis speed, as well as ultra-low power consumption [7].

The resonant microcantilever employed in MEMS TGA conserves mechanical energy during resonance, generating a frequency-shift signal that reflects real-time mass changes [13,14]. To achieve this, a resistive microheater positioned near the cantilever’s free end is utilized to heat the sample, reaching temperatures of up to 1200 °C. By controlling the heating current using a precalibrated metal heating resistor as a thermometer, precise temperature control is achieved. The mass variation during the controlled heating process is measured using a piezoresistive Wheatstone bridge, providing a real-time reading of the frequency-shift signal. This system integrates piezoresistive sensing elements and thermoelectric resonance excitation elements on the cantilever and maintains resonance using a phase-locked loop interface circuit.

However, using a resonant microcantilever as the measuring core in the MEMS TGA has some drawbacks. Firstly, the mass sensitivity of the microcantilever varies significantly along its length, with a several-fold difference between the sensitivity at the free end and the middle position [15]. In general, the coating location of the test sample is near the free end of the microcantilever, which cannot be equivalently represented as a single-point mass. The calibration of mass sensitivity often only involves the free end position of the microcantilever, which cannot represent the average sensitivity at the actual sample coating position. The calculation of mass change is based on the sensitivity and resonant frequency shift. Inaccurate sensitivity will therefore lead to inaccurate measurements of mass changes. Secondly, the temperature distribution in the sample area of the microcantilever is also not uniform. Because the heat sink of the microcantilever is at the fixed end of the microcantilever, the temperature gradually decreases from the free end to the fixed end along the microcantilever [16]. The non-uniform temperature distribution of the microcantilever will affect the temperature accuracy of TGA testing.

Herein, a specifically designed integrated H-shaped resonant beam is proposed and developed to serve as an ultra-sensitive micro-thermogravimetric chip for MEMS TGA characterization and bio/chemical sensing. Through the optimized design of the resonant beam shape and the layout of the integrated heating resistor, significant improvements were achieved in the mass sensitivity and temperature distribution uniformity of the resonant beam compared to the microcantilever. These improvements were validated through characterizations using laser Doppler and infrared spectroscopy. Gas-sensing and TGA experiments using the resonant beam were conducted, yielding satisfactory test results.

## 2. Design

### 2.1. Structural Design

**Figure 1:**
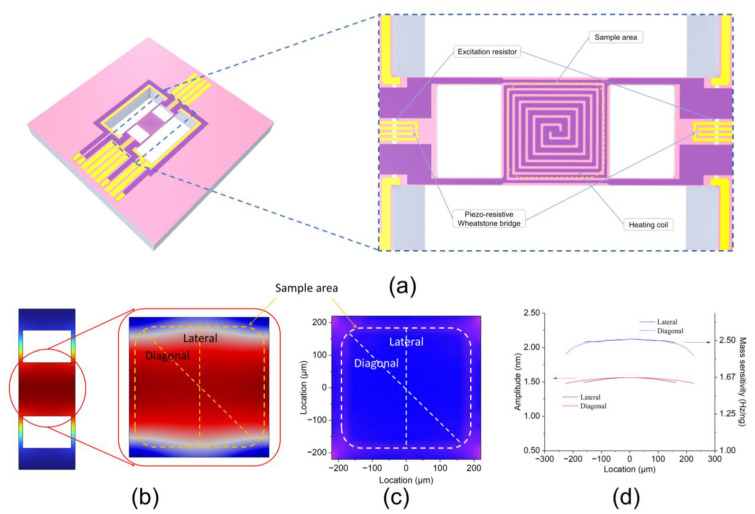
Structural diagram and FEI simulation results: (**a**) structural diagram of the H-shaped resonant beam; (**b**) FEI simulation results of the amplitude distribution of the beam; (**c**) FEI simulation results of the mass sensitivity distribution of the beam; (**d**) distribution values of amplitude and mass sensitivity in the lateral and diagonal directions of the sample area.

Due to the positive correlation between the mass sensitivity at different positions of the resonant beam and the amplitude at those positions [15], we designed an H-shaped resonant beam, shown in Figure 1a, to ensure uniform mass sensitivity. The resonant beam has three zones: a high-temperature zone in the middle and a low-temperature zone on each side. The high-temperature zone and the low-temperature zones are connected through four small beams. The high-temperature zone contains embedded heating coils, which are used to heat the sample area located within it and simultaneously measure the temperature through its resistance. The resonance excitation and frequency detection elements are integrated into the low-temperature zone to mitigate the influence of temperature. We utilized finite element analysis (FEA) software COMSOL Multiphysics 6.0 to characterize the distributions of amplitude and mass sensitivity. Mass sensitivity is defined as the change in resonant frequency after applying the mass point divided by the mass applied, and it can be simulated by applying mass points at different positions in the sample area. Figure 1b shows the amplitude simulation results of the resonant beam at various positions, and Figure 1c shows the mass sensitivity simulation results of the sample area. From the figure, it can be observed that the overall displacement of the high-temperature zone approximates an up-and-down vibration pattern, with slightly larger amplitude deviations at the four diagonal corners due to the connecting small beams. The curves in Figure 1d quantitatively describe the amplitude and mass sensitivity values obtained along the lateral and diagonal directions in the sample area. From these curves, it can be calculated that the maximum percentage difference between the amplitudes at the center and the lateral edge in the sample area is 5.3%, and the difference with the diagonal edge is similar. As a comparison, the amplitude difference percentage in the sample area of the microcantilever presented in the literature [7] exceeds 50%.

### 2.2. Heating Design

**Figure 2:**
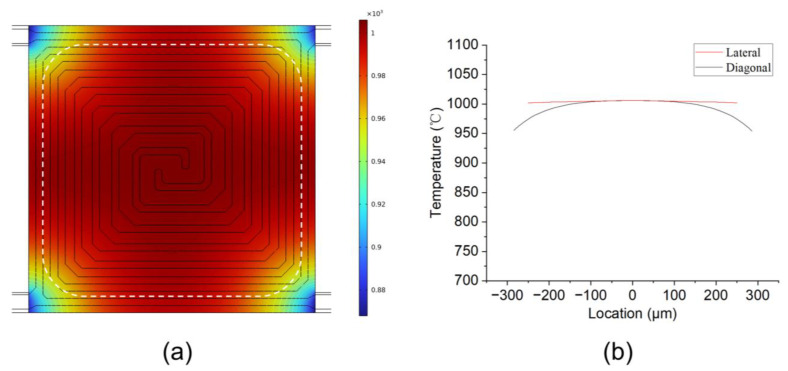
(**a**) FEI simulation results of the temperature distribution of the resonant beam (Circled part is the sample area); (**b**) temperature distribution values in the lateral and diagonal directions of the sample area.

The uniformity of temperature has a significant impact on TGA testing. Due to the heat sinks located at the four corners of the resonant beam sample area, heat tends to diffuse from the center toward the corners. In order to improve temperature uniformity, the peripheral heating power should be higher than the central heating power. To achieve this goal, the microheater was designed in a spiraling shape, with the width of the heater gradually increasing from the edge to the center of the sample area, as shown in Figure 2a. From the FEA simulation results shown in Figure 2, it can be observed that the temperature uniformity in the majority of the sample area is above 95%. Molybdenum (Mo) was chosen as the material of the heater, as Mo has high electron density, a linear TCR (temperature coefficient of resistance), and a high melting point [17,18]. Moreover, the temperature of the molybdenum heater can be accurately controlled using a closed-loop feedback system comprising four probes. Two pads were used to supply heating current, while the other two pads measured resistance [19,20]. However, although Mo has an exceptionally high melting point of about 2620 °C, it is easily oxidized. The reaction between Mo and O_2_ at different temperatures results in different compounds. At room temperature, the reaction between Mo and O_2_ is slow. However, at temperatures ranging from 300 °C to 600 °C, the reaction accelerates, and a MoO_2_ oxide film begins to form on the surface. Above 600 °C, the primary product formed is MoO_3_, which is a yellowish solid. At high temperatures, MoO_3_ can also form a composite with Mo, known as MoO_3_-MoO_2_, or react further with O_2_ to produce compounds of higher oxidation states. Thus, the Mo surface needs to be coated with a protective layer. This protective layer not only prevents oxidation but also provides electrical insulation. In general, silicon nitride is a good choice as the protective layer.

### 2.3. Excitation and Detection

There are various excitation methods for resonant beams, such as thermoelectric excitation [21,22], electromagnetic excitation [23], electrostatic excitation [24], and piezoelectric excitation [25]. Frequency detection methods include capacitance detection [26], piezoelectric detection [27], resistive detection [28], and optical detection [29]. In this work, the resonant beam underwent thermoelectric excitation by integrating resistors near each fixed end of the beam, and the frequency signals were detected with a piezoresistive Wheatstone bridge. Both the piezoresistive sensing elements and thermoelectric resonance exciting elements were integrated in the low-temperature zone, where the temperature remained below 120 °C even when the high-temperature zone was heated to 1000 °C. This could effectively avoid the failure of the exciting and detecting resistors due to high temperatures when the sample area was heated.

## 3. Fabrication of the Resonant Beam

**Figure 3:**
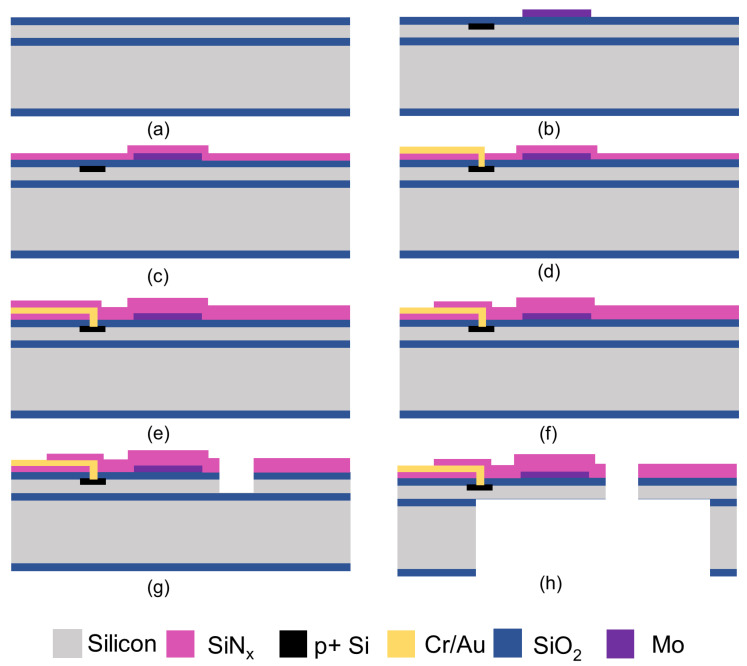
Fabrication processes of the resonant beam: (**a**) oxidation of the SOI wafer; (**b**) ion implantation and heating coil patterning; (**c**) passivation layer growth; (**d**) contact holes’ opening and metal leads’ patterning; (**e**) growth of the second passivation layer; (**f**) passivation layer patterning; (**g**) resonant beam outline shaping; (**h**) release of the resonant beam.

The fabrication process of the resonant beams is divided into eight steps, as shown in Figure 3. First, 4-inch SOI silicon wafers with 10 μm device layer, 1 μm BOX layer, and 400 μm handle layer were used to fabricate the resonant beams. The fabrication process mainly involved two key aspects: the construction of the suspended structure and the formation and the protection of the heating metal coil. Using SOI wafers allowed for the precise control of the thickness of the suspended structure. The release of the suspended structure was achieved through double-sided DRIE (deep reactive ion etching). The Mo metal coil was fabricated using a lift-off technology. The dimensions of the Mo metal coil need to be precisely controlled to ensure uniform heating. The post-metal protection film was fabricated using PECVD (plasma-enhanced chemical vapor deposition), which is a low-temperature process.

The detailed fabrication processes are listed below.

(a) First, 300 nm SiO_2_ was thermally oxidized on the wafer as a passivation layer. Reactive ion etching (RIE) technology was used to create the alignment marks.
(b) The exciting resistors and piezoresistive Wheatstone bridge were created by boron ion implantation, with patterned photoresist as a mask. Then, Mo resistors with 100 nm thickness were sputtered and patterned using lift-off technology.
(c) A 100 nm thick SiN_x_ film was deposited via plasma-enhanced chemical vapor deposition (PECVD) for electric passivation.
(d) RIE technology was employed to open the contact holes. After that, a 330 nm thick Cr/Au composite layer was sputtered and patterned using lift-off technology.
(e) Another 150 nm thick SiN_x_ film was deposited by PECVD for passivation from the external environment.
(f) RIE technology was used to pattern the passivation layer grown in the previous step.
(g) RIE technology was used to selectively remove the three passivation layers until the underlying silicon layer was exposed, and then the exposed silicon layer was etched using deep RIE technology to shape the resonant beam outline.
(h) The wafer was etched from the backside using deep RIE to remove the silicon beneath the beam. After that, the BOX layer was removed via BOE (buffered oxide etch) wet etching.

Finally, the wafer was diced with a laser saw (Disco DFL7341) into chips.

## 4. Characterization and Experimental Results

### 4.1. Mass Sensitivity Test

**Figure 4:**
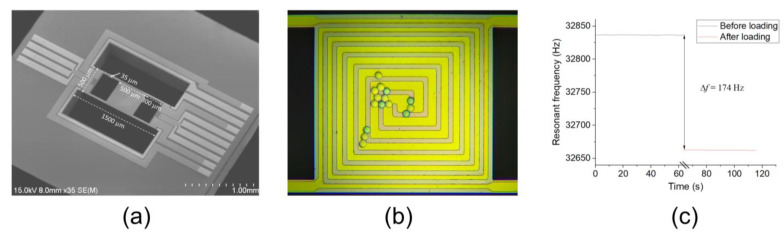
(**a**) SEM image of the H-shaped resonant beam; (**b**) optical photos of the resonant beam with PS spheres loaded; (**c**) curve of resonant frequencies before and after PS spheres were loaded.

The SEM image of the fabricated resonant beam chip is shown in Figure 4a, with the dimensions of the main parts annotated. The length, width, and thickness of the H-shaped beam were 1500 μm, 500 μm, and 10 μm, respectively. The size of the square area in the center was 500 μm × 500 μm. There are two reasons for choosing these dimensions of the beam. Firstly, the resonant frequency of the resonant beam was around 30 kHz, which is suitable for electrothermal excitation [30]. Secondly, the sample area was large enough for sample loading.

Equipped with a phase-lock-looped interface circuit [30], the resonant beam was maintained in the resonant state, and the resonant frequency could be directly read. First, we used polystyrene (PS) spheres to measure the sensitivity of the resonant beam. As shown in Figure 4b, 15 PS spheres with diameters of 20 μm were loaded onto the sample area of the resonant beam using a commercial Microchip Spotter (Xiamen High-End MEMS Technology Co., Ltd., Xiamen, China; model: LoC-MS 1000). Each sphere had a mass of 4.4 ng, with a total loading mass of 66 ng. The resonant frequency of the resonant beam changed by 174 Hz before and after loading PS spheres, as shown in Figure 4c. The mass sensitivity of the resonant beam was calculated as 2.64 Hz/ng, as can be seen from Figure 4c, which was very close to the simulated value of 2.49 Hz/ng.

### 4.2. Amplitude and Sensitivity Distribution Tests

**Figure 5:**
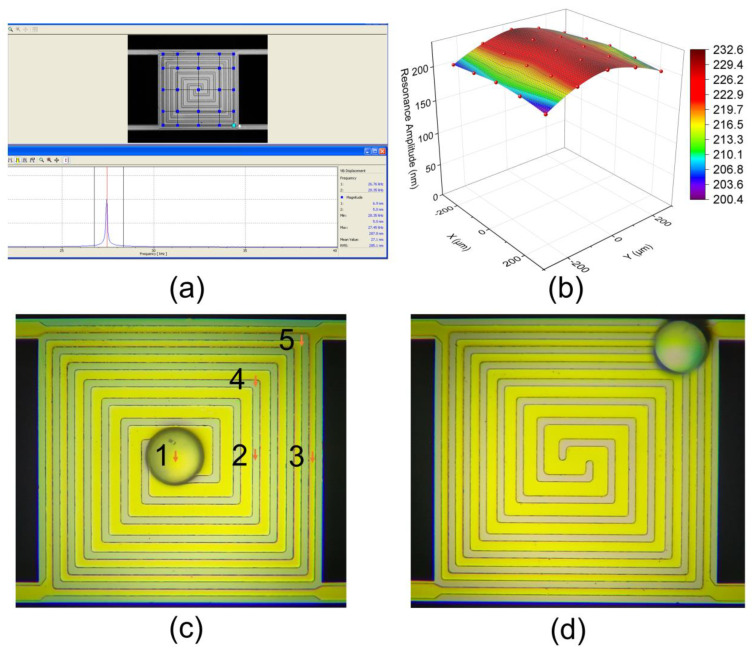
(**a**) Test interface of amplitude distribution with a laser Doppler analyzer; (**b**) test results of the amplitude distribution; (**c**,**d**) optical images of the resonant beam sample area with PS spheres loaded at different locations.

To verify the proposed resonant beam’s consistency in mass sensitivity, amplitude and sensitivity distribution tests were conducted. The amplitude distribution test on the resonant beam sample area was conducted using a laser Doppler vibrometer (Polytec GmbH, Waldbronn, Germany; model: MSA500). After the resonant beam was excited into a resonant state, we used the laser Doppler vibrometer to test the amplitude of 25 positions in the sample area, as shown in Figure 5a. The amplitude distribution results are shown in Figure 5b and listed in Table 1. Based on the test results, it can be observed that the amplitude of the resonant beam was highest at the middle positions and gradually decreased toward both sides along the length direction. The amplitude distribution uniformity (defined as (A_max_ − A_min_)/A_avg_ × 100%) was around 14.7%, which matched the simulated values. It is worth noting that under normal usage, samples are generally not loaded onto the four corner positions. If these positions are excluded, the uniformity will be improved to 11.7%.

To further test the distribution of mass sensitivity, we conducted tests using a PS sphere with a diameter of 80 μm. The reason for using such a large PS sphere is that it can be easily pushed with a capillary to reach different positions of the resonant beam. By moving it to five different positions, as shown in Figure 5c, we obtained the resonant frequency changes before and after placing the sphere in different positions, which were further calculated as mass sensitivity values, and the results are listed in Table 2. The distribution of mass sensitivity was similar to the amplitude distribution, with higher sensitivity in the middle and lower sensitivity toward the edges. The mass sensitivity distribution was uniform across the vast majority of sample areas, with a standard deviation of 0.15. The maximum difference occurred in the corners outside the sample area, with a difference rate of 14.9%.

### 4.3. Temperature Uniformity

**Figure 6:**
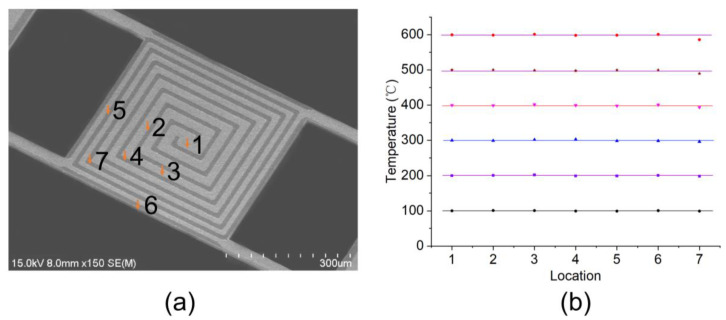
Test of temperature distribution: (**a**) SEM image of the resonant beam sample area with marked test positions; (**b**) temperature measurement results at these positions under a series of set temperatures ranging from 100 °C to 600 °C.

Temperature uniformity is an important parameter of resonant beams, as it affects the simultaneous occurrence of sample reactions in TGA testing. To achieve temperature control, the first step is to measure the TCR of the heating coil. The four-probe method was employed to measure the resistance of the metal heating coil [19]. The temperature measurements were obtained using an infrared thermometer. The measured TCR value was determined to be 0.002323 °C^−1^, and remained consistent over a broad temperature range. This uniformity facilitated precise temperature regulation through closed-loop feedback mechanisms. The temperature measurements were performed on different locations of the resonant beam sample area, as shown in Figure 6a. When the sample area was heated to the set temperatures ranging from 100 °C to 600 °C, temperatures at the seven locations were measured, as shown in Figure 6b. Close to the simulated results in Figure 2, the temperatures at positions 1–6 were essentially the same. The temperatures at position 7 were slightly lower. The measured temperature difference between different positions in the sample area was calculated within 10%, demonstrating that the resonant beam had good temperature uniformity.

### 4.4. TGA and Gas-Sensing Application Tests

**Figure 7:**
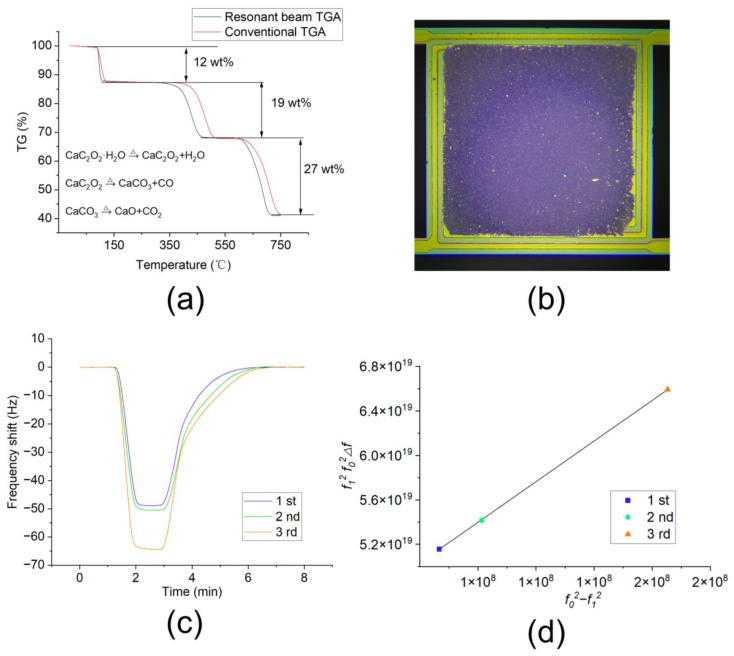
(**a**) TGA test results of the resonant beam; (**b**) optical images of the resonant beam with MOF material loaded; (**c**) gas-sensing results of the resonant beam; (**d**) normalized results.

TGA and gas-sensing tests were executed to validate the performance. The TGA test results of calcium oxalate monohydrate (CaC_2_O_4_·H_2_O) using the resonant beam and conventional TGA instrument are shown in Figure 7a. CaC_2_O_4_·H_2_O undergoes three decomposition stages at distinct temperatures, yielding three volatile products: H_2_O, CO, and CO_2_. Each stage of decomposition is accompanied by a decrease in mass as the volatile products are released. From the comparison of the TGA curves in the figure, it can be observed that the weight loss in the three stages between the resonant beam TGA and conventional TGA are almost identical. The resonant beam TGA shows slightly lower temperatures for each stage. This is because the resonant beam TGA can effectively eliminate the thermal hysteresis effect [7]. Compared with the MEMS cantilever TGA curve in the literature [7], the resonant beam TGA shows a smoother TGA curve, which benefits significantly from the improved temperature uniformity.

In order to verify the uniformity of mass sensitivity distribution, a gas-sensing experiment was executed. Due to the proportional relationship between the gas-sensing response signal and the coating amount of sensitive material, in order to compare the sensor responses with different loading amounts of the sensing material, it is necessary to normalize the response signal. For a resonant beam without any material loaded, the resonant frequency *f*_0_ is as follows:

(1) $$f_{0}=\frac{1}{2\pi }\sqrt{\frac{k}{m_{eff}}}$$

where *k* is the elastic modulus, and *m*_eff_ is the effective mass of the beam.

After loading the sensing material, the mass change in the resonant beam ∆m (i.e., the mass of the sensing material loaded) can be represented as follows:

(2) $$∆m=\frac{k}{4\pi^{2}}(\frac{1}{{f_{1}}^{2}}-\frac{1}{{f_{0}}^{2}})\;$$

where *f*_1_ is the resonant frequency after the sensing material is loaded.

The sensitivity of the resonant beam is as follows:

(3) $$S=\frac{∆f}{∆m}$$

where ∆f is the resonant frequency shift signal after the target gas adsorption occurs.

Then, we have the following:

(4) $$S=\frac{4\pi^{2}}{k}\frac{f_{1}^{2}f_{0}^{2}∆f}{f_{0}^{2}-f_{1}^{2}}\;$$

Equation (4) means that, for a resonant beam with uniform sensitivity, after coating different amounts of sensitive material, the response values to the target gas at identical concentrations must meet the following equation:

(5) $$f_{1}^{2}f_{0}^{2}∆f\;\propto f_{0}^{2}-f_{1}^{2}\;$$

Equation (5) can be used to examine whether the sensitivity distribution of a resonant beam is uniform. The operational method is as follows:

First, test the resonant frequency *f*_0_ of a resonant beam without any material loaded. Then load the sensing material and conduct gas-sensing testing to obtain *f*_1_ and ∆f. After that, load more sensing material to the resonant beam and conduct gas-sensing testing. Then repeat the above process. Now that we have three sets of data, we can check if they satisfy the proportional relationship in Equation (5).

Herein, a type of Cu-based metal–organic framework (MOF) material composed of HHTP(2,3,6,7,10,11-hexahydroxytriphenylene) and Cu metal center was used to detect ammonia (NH_3_) gas. First, an appropriate amount of Cu-based MOF material was added to 1 mL of deionized water (under ultrasonic) to form a crude suspension. Then, about 1 μL of the suspension was loaded onto the sample area of the resonant beam using a micropipette with the aid of a microscope. Finally, the resonant beam was dried in an oven at 60 °C for about 2 h. The resonant beam with the MOF material loaded is shown in Figure 7b.

The gas-sensing experiments were performed at room temperature with the help of an Intelligent Dynamic Gas Distribution System (Xiamen High-End MEMS Technology Co., Ltd., Xiamen, China; model: LoC-GDS 4000). The resonant beam sensor was placed in a closed chamber, which was sequentially filled with nitrogen gas, ammonia gas with a concentration of 100 ppm, and nitrogen gas. After each sensing experiment, an appropriate amount of Cu-based MOF material was added onto the same resonant beam sensor using the abovementioned sample loading method, and the gas-sensing experiment was repeated. The NH_3_ gas-sensing response signals of the same resonant beam with different amounts of sensing material are shown in Figure 7c. These gas-sensing response signals were normalized according to Equation (5), and the obtained curve is shown in Figure 7d. From the figure, it is evident that this is a linear curve. This proves that the sensitivity of the resonant beam remained unchanged after loading different amounts of the sensitive material, thereby verifying its uniform sensitivity distribution.

## 5. Conclusions

In this study, we proposed and developed an H-shaped resonant beam based on MEMS technology for gas-sensing and TGA applications. Compared with resonant cantilevers, the resonant beam was designed with much better performance in the distribution of both temperature and sensitivity. The resonant beam was characterized and tested, and the results showed that the uniformity of mass sensitivity was within 15%, while the uniformity of temperature distribution was below 10%. TGA and gas-sensing tests were conducted, and the test results verified its advantage in uniformity. This resonant beam with outstanding sensitivity and temperature uniformity has enormous potential for various gravimetric characterizations.

## Figures and Tables

**Table 1:** Amplitude distribution data obtained with a laser Doppler analyzer.

Amplitude (nm)	1	2	3	4	5
1	203.8	222.8	232.6	224.2	206.7
2	206.7	221.8	230.0	223.3	210.3
3	209.4	219.4	228.5	222.8	213.2
4	206.8	220.5	229.5	222.7	209.2
5	200.4	222.1	231.3	222.5	205.0

**Table 2:** Resonant frequency changes and calculated mass sensitivity data at different positions obtained by loading a PS sphere.

Positions	1	2	3	4	5
Resonant frequency change (Hz)	744.5	743.4	743.1	702.6	633.6
Mass sensitivity (Hz/pg)	2.64	2.64	2.64	2.50	2.25

## Data Availability

The data presented in this study are available upon request from the corresponding author.
